# Ligand-Mediated, Temperature-Tuned Synthesis of CsPbBr_3_ Nanosheets for Ordered Superlattice Assembly

**DOI:** 10.3390/ma18214885

**Published:** 2025-10-24

**Authors:** Zahir Abdalla, Chengqi Liu, Shefiu Kareem, Xiaoqian Wang, Zisheng Tang, Yong Liu

**Affiliations:** State Key Laboratory of Advanced Technology for Materials Synthesis and Processing, Wuhan University of Technology, Wuhan 430070, China; zahiralbashir3@gmail.com (Z.A.); liuchengqi42@163.com (C.L.); shefiu548@hotmail.com (S.K.); 303568@whut.edu.cn (X.W.); tangzs3076@163.com (Z.T.)

**Keywords:** CsPbBr_3_ NSs, photoluminescence, superlattice, quantum confinement, self-assembly

## Abstract

Two-dimensional (2D) colloidal CsPbBr_3_ nanosheets (NSs) possess size-dependent optoelectronic properties; however, conventional hot-injection methods often lack precise growth control and well-ordered superlattice self-assembly. Herein, we introduce a modified ligand-assisted hot-injection strategy that promotes direct precursor–ligand interactions prior to solvent mixing, thereby enabling highly controlled nanosheet superlattice growth. By adjusting the reaction temperature from 130 to 150 °C, we obtained rectangular nanosheets with monodisperse, well-defined thicknesses of 3.35 ± 0.05 nm and 4.05 ± 0.09 nm at 130 and 140 °C, respectively, both below the 7 nm exciton Bohr diameter, consistent with strong quantum confinement. The resulting superlattices exhibited sharp, tunable photoluminescence peaks at 462, 464, and 513 nm, with time-resolved PL revealing a clear size–lifetime correlation, where smaller lateral superlattices at 130 °C showed a short decay time of 8.65 ns, intermediate growth at 140 °C yielded 15.42 ns, and larger lateral superlattices at 150 °C reached 35.49 ns. Importantly, the modified synthesis facilitated the formation of ordered superlattices that preserved their intrinsic emission properties, underscoring their structural stability and scalability. These findings establish a direct link between ligand-mediated synthesis, reaction temperature, nanosheet dimensions, and optical performance, offering a pathway to high-quality perovskite NS superlattices for advanced optoelectronic applications such as light-emitting diodes and sensors.

## 1. Introduction

Perovskite nanostructures have emerged as a transformative class of materials owing to their exceptional optical and electronic properties, including high photoluminescence quantum yields, tunable bandgaps, and robust exciton coupling [1,2,3,4,5]. Their structural versatility and solution processability make them promising candidates for next-generation optoelectronic devices [6,7,8,9]. These nanostructures can be synthesized in diverse morphologies, such as nanosheets, nanospheres, nanorods, and nanowires [10,11,12]. Their shape and size can be tuned by varying the synthetic conditions, including temperature, ligand composition, and reaction duration. Methods such as hot-injection, room-temperature synthesis, and two-step growth have been successfully employed to produce tailored morphologies [2,13,14], while other strategies exploit natural growth patterns to obtain uniform structures, such as nanoplates and nanowires [15,16,17]. In particular, nanosheets have attracted attention due to their ultrathin morphology, which enhances light–matter interactions and enables applications in advanced displays, sensors, and quantum devices.

Among these nanostructures, two-dimensional (2D) colloidal nanosheets are of special interest because their strong quantum confinement effects yield size-dependent properties that are highly attractive for optoelectronics, quantum technologies, and chiroptical applications [18,19]. Several reliable colloidal synthesis techniques have been reported for the preparation of high-quality 2D CsPbX_3_ nanosheets. For example, Shamsi et al. [18] tuned the lateral dimensions of CsPbBr_3_ nanosheets by adjusting the ratio of short ligands (octanoic acid and octylamine) to long ones (oleic acid and oleylamine). Liang et al. [20] employed carefully measured oleic acid and octylamine ligands to synthesize nanosheets with an average lateral size of 75 nm, while Sun et al. [10] achieved 5.2 nm-thick nanosheets with lateral dimensions of ~100 nm by replacing hexanoic acid with oleic acid. Bekenstein et al. [21] reported the formation of 2D CsPbBr_3_ nanosheets with thicknesses down to 3 nm through a directional (oriented) attachment process, where small single-crystal plates align and merge laterally along specific crystallographic directions while ligand binding restricts vertical growth. These studies highlight the significant impact of organic ligands on the lateral size of CsPbBr_3_ nanosheets, yet the mechanistic link between ligand characteristics and lateral dimensions remains poorly understood. Furthermore, the assembly of single plates into 2D rather than 3D structures is not fully defined, although such mechanistic insights are essential for rationally designing nanosheets with tailored dimensions. The ultrathin morphology of the CsPbBr_3_ nanosheets, with thicknesses comparable to or smaller than the exciton Bohr diameter (~7 nm), induces strong quantum and dielectric confinement, thereby increasing the exciton binding energy by one to two orders of magnitude relative to the bulk [12,21,22]. This reduced dimensionality also leads to anisotropic optical responses, which are advantageous for polarized-light detection [23].

The self-assembly of nanocrystals is governed by a delicate balance between repulsive and attractive interactions, including steric repulsion from surface ligands and electrostatic or van der Waals attractions between particles [24,25,26]. For 2D nanostructures, such as nanosheets and nanoplates, ligand–surface interactions play a dominant role compared to 1D nanostructures, such as nanorods or nanowires, because of the higher surface area available for ligand binding [27,28,29]. Solution-based crystallization remains the most common route for preparing 2D layered perovskites such as CsPbBr_3_ nanosheets [30,31]. However, successful self-assembly requires both intrinsic nanocrystal properties and external driving forces that promote ordering [31,32]. Factors such as nanocrystal size distribution, concentration, and shape strongly influence the assembly outcome [24,33]. Ordered arrays of nanowires, nanosheets, or even three-dimensional (3D) crystals can be achieved when monodisperse nanocrystals are employed [34]. For nanosheets, vertical layer-by-layer stacking is commonly observed during solvent evaporation, whereas both vertical and horizontal arrangements of nanoplatelets can occur in solution depending on nanocrystal dimensions and medium [35]. Although methods such as slow cooling or rapid solvent evaporation can promote ordering, they often lack precise control over the final nanosheet morphology, internal structure, and nanoscale composition [36].

To address these challenges, we employed an improved hot-injection strategy to synthesize 2D CsPbBr_3_ nanosheets with finely controlled thicknesses. These nanosheets readily stack into well-ordered superlattices through a self-assembly process, significantly enhancing their optoelectronic properties. We further investigated their shape, structural characteristics, optical properties, and assembly behavior, providing new mechanistic insights into the design of 2D CsPbBr_3_ nanosheets for advanced optoelectronic and photoelectric applications.

## 2. Materials and Methods

### 2.1. Materials

Cesium Carbonate (Cs_2_CO_3_, 99.9%), Lead Bromide (PbBr_2_, 99.999% trace metals basis), Octylamine (OctAm, 99%), 1-octadecene (ODE, 90% technical grade), octanoic acid (OctAc, 99%), and oleylamine (OAm, 80-90%) Oleic acid (OA, 90%) and hexane (99%) were obtained from Aladdin, Shanghai, China. All Chemicals were used as received without further purification.

### 2.2. Methods

#### 2.2.1. Cesium Oleate Precursor Synthesis

A typical synthesis was carried out as follows: 0.10 g of Cs_2_CO_3_ (0.307 mmol) and 10 mL of oleic acid (OA) were loaded into a 50 mL three-neck round-bottom flask. The mixture was then degassed and dried under vacuum at an oil bath temperature of 115 °C for 2 h with constant stirring. After drying, the mixture was heated in an oil bath at 150 °C under a nitrogen atmosphere until cesium carbonate completely reacted with oleic acid, yielding a Cs-oleate concentration of 0.031 M. The cesium oleate solution was stored and used for the subsequent synthesis of CsPbBr3 nanosheets.

#### 2.2.2. Synthesis of CsPbBr_3_ Nanosheets

**Figure 1:**
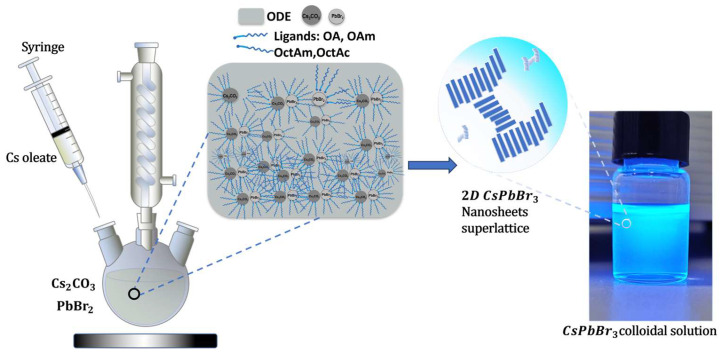
Schematic diagram of the ligand-modified synthesis procedure for CsPbBr_3_ NSs superlattices.

CsPbBr_3_ nanosheets were synthesized following a modified procedure reported by Yong et al. [32] In a typical synthesis, 0.15 g of PbBr_2_ (0.4087 mmol) and 1 mL of oleic acid were loaded into a 50 mL three-neck round-bottom flask and heated to 115 °C under nitrogen atmosphere. A ligand mixture consisting of 10 mL of octadecene (ODE), 0.25 mL of oleylamine (OAm), 0.75 mL of octylamine (OctAm), and 0.75 mL of octanoic acid (OctAc) was then injected into the flask while maintaining the temperature at 115 °C. After ligand injection, the mixture was degassed under vacuum at 115 °C for 10 min to remove residual moisture and oxygen, following established CsPbBr_3_ synthesis protocols [2,10,18]. No visual changes or degradation of the PbBr_2_ ligand complexes were observed during this step. The solution was then heated to 130–150 °C. At the target temperature, 0.50 mL of a 0.031M pre-prepared Cs-oleate solution was swiftly injected into the reaction mixture under constant stirring. The reaction was immediately indicated by a rapid color change in the solution, which was confirmed by bright blue photoluminescence under 365 nm UV illumination. After 30 s, the reaction was quenched immediately by immersing the flask in an ice water bath, yielding uniform nanosheet superlattices (Figure 1 and Figure S1a).

#### 2.2.3. Isolation and Purification of Nanosheets

To isolate the CsPbBr_3_ nanosheets, 10 mL of n-hexane was added to the crude nanosheet superlattice solution to facilitate the precipitation. The suspension was centrifuged at 10,000 rpm for 10 min, and the process was repeated once with an additional 5 min run to ensure the complete separation of nanosheets from unreacted precursors and by-products. The precipitate was redispersed in 5 mL of n-hexane and centrifuged again at a lower speed (500–1000 rpm for 5 min) to remove residual impurities. The resulting supernatant containing the purified nanosheets was collected and transferred to clean scintillation vials. The purified nanosheets were subsequently used for further characterizations, such as transmission electron microscopy (TEM), photoluminescence (PL), UV-Vis absorption, and time-resolved photoluminescence measurements.

### 2.3. Characterization

#### 2.3.1. Characterization of Material Morphology

Transmission electron microscopy (TEM) analysis was performed using a JEOL JEM-1400 Plus instrument (JEOL Ltd., Tokyo, Japan) operated at 120 kV with a thermionic electron source. For sample preparation, colloidal nanosheet dispersions were deposited onto 300-mesh copper grids coated with an ultrathin amorphous carbon support film. High-angle annular dark-field scanning transmission electron microscopy (HAADF-STEM) imaging was performed, and elemental analysis was conducted using an energy-dispersive X-ray (EDX) detector integrated with the TEM. High-angle annular dark-field scanning TEM (HAADF-STEM) images were obtained using a Talos F200S-type field emission transmission electron microscope (Thermo Fisher Scientific, Hillsboro, OR, USA) operated at 200 kV. The loaded grids were subsequently dried under high vacuum to ensure complete solvent removal and minimize the oxidation of the nanosheets. The lateral size and thickness of the nanosheets were evaluated using statistical measurements from the TEM images using Nano Measurer software (version 1.2). More than 50 individual nanosheets were analyzed to obtain reliable distribution profiles of the elements.

#### 2.3.2. Structural and Surface Characterization

X-ray Diffraction (XRD) measurements were performed using a Bruker D8 diffractometer with Cu Kα radiation (λ = 1.54056 Å) operated at 40 kV and 40 mA. A diluted suspension of perovskite nanosheets in n-hexane was drop-cast onto SiO_2_/Si substrates and dried before analysis. X-ray Photoelectron Spectroscopy (XPS) analysis of the surface composition and chemical states was carried out using a Thermo Scientific Kα spectrometer equipped with a monochromatic Al Kα source (hν = 1486.68 eV). The base pressure in the analysis chamber was maintained at 5 × 10^−10^ Pa. The operating voltage and filament current were set to 15 kV and 10 mA, respectively. Signal accumulation was performed for 5–10 cycles. The pass energy was set to 50 eV, with a step size of 0.1 eV. All spectra were calibrated by referencing the adventitious carbon C 1s peak at 284.8 eV (C–C bonds) to compensate for surface charging [37,38]. Fourier-transform infrared (FTIR) spectroscopy measurements were performed using a Nicolet 6700 spectrometer (Thermo Fisher Scientific, Waltham, MA, USA). Spectra were collected over the range of 400–4000 cm^−1^ with a spectral resolution of 4 cm^−1^ and an acquisition rate of one scan per second. The spectrometer employs a frequency-modulation interferometer to generate an interferometric light signal, which is processed into spectra through Fourier transformation using the OMNIC 3.0 software package after interaction with the sample. For sample preparation, concentrated nanosheet dispersions were drop-cast onto potassium bromide (KBr) wafers and dried before measurement.

#### 2.3.3. Fluorescence Spectrum Characterization

UV–Vis absorption spectra were recorded using a Shimadzu UV-1800 spectrophotometer (Kyoto, Japan) with UVProbe 2.52 software. Steady-state photoluminescence (PL) spectra were obtained using a Shimadzu RF-6000 spectrofluorometer (Kyoto, Japan) operated with the built-in LabSolutions RF software provided by the manufacturer, with an excitation wavelength (λex) of 400 nm. Perovskite nanosheet (NS) samples were prepared by diluting the NS solution in n-hexane and placing it in quartz cuvettes with a 10 mm path length. Time-resolved photoluminescence (TRPL) measurements were performed by monitoring the emissions at 462, 464, and 513 nm, corresponding to the respective PL peaks of the nanosheets, using a 405 nm pulsed diode laser for the blue-emitting samples and a 485 nm pulsed diode laser for the green-emitting sample (pulse width of 200 ps and pulse energy of 14 pJ; NanoLED-C2 series, HORIBA Scientific, Kyoto, Japan).

## 3. Results

Previous reports from our research group have demonstrated the synthesis of CsPbBr_3_ nanosheet superlattices using hot-injection strategies; however, these approaches often lack precise control over nanosheet dimensions and the reproducibility of self-assembly [12,32]. In the conventional ODE-first method, oleic acid (OA) does not strongly bind to the precursors during the early reaction stage, leading to irregular regulation of crystal growth and partial loss of OA during purification of the nanocrystals. This weak binding not only disrupts nucleation and growth control but also diminishes the ligand’s role in supporting the self-assembly of the nanosheets.

In contrast, our modified ligand-assisted protocol employs a pre-mixing step in which OA is first combined with the precursors before the addition of the solvent. This ensures strong precursor–ligand interactions that stabilize nucleation and direct the growth pathway, yielding nanosheets with uniform lateral dimensions and thicknesses. Moreover, because OA is well integrated from the outset, it remains effective during purification, supporting the layer-by-layer self-assembly of nanosheets into an ordered superlattice. Oleic acid facilitates the self-assembly of nanocrystals by stabilizing their surfaces and has been employed in post-synthetic treatments to promote the formation of ordered halide perovskite superlattices [39]. By systematically tuning the reaction temperature, we achieved reproducible control over the nanosheet morphology and optoelectronic properties, thereby establishing a direct correlation between the synthesis conditions, reaction temperature, ligand dynamics, and superlattice formation.

**Figure 2:**
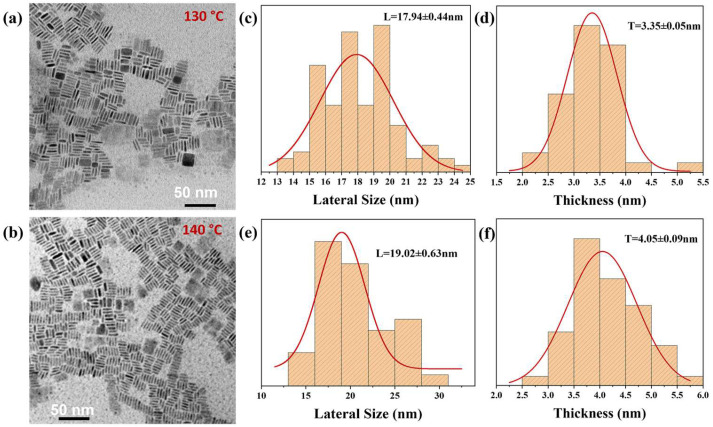
(**a**) TEM image of CsPbBr_3_ NSs at 130 °C, (**c**,**d**) size distribution at 130 °C, (**c**) lateral size, and (**d**) thickness. (**b**) TEM image of 2D CsPbBr3 NSs at 140 °C, (**e**,**f**) size distribution at 140 °C, (**e**) lateral size, and (**f**) thickness.

**Figure 3:**
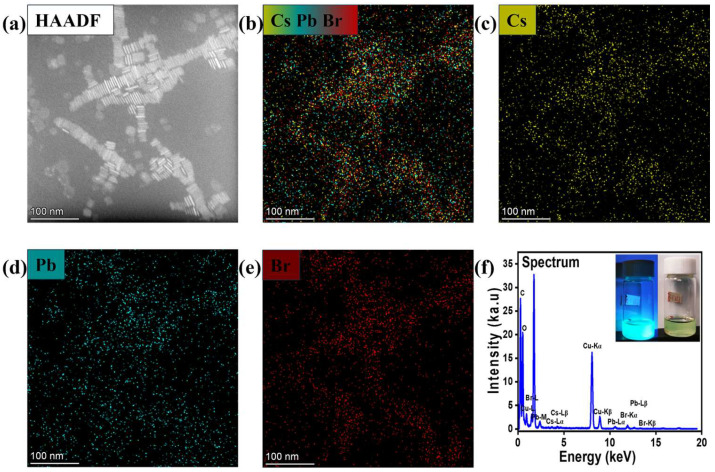
(**a**–**e**) High-resolution dark-field TEM image. (**a**) Corresponding elemental mapping images of CsPbBr_3_ NSs (**b**) Combination of Cs, Pb, and Br (**c**), Cs (**d**), Pb (**e**), and Br (**f**) Elemental spectra patterns for the CsPbBr_3_ nanosheet superlattice prepared at 140 °C. Insert is CsPbBr_3_ NSs superlattice under 365 nm UV illumination and under ambient light.

Under these optimized conditions, the synthesis naturally led to the formation of self-assembled nanosheet superlattices with tunable structural and optical characteristics. The resulting assemblies consisted of nanosheets with thicknesses ranging from 3.35 to 6.5 nm and lateral sizes increasing from 17.9 to 242.8 nm as the synthesis temperature increased. These nanosheets formed well-ordered, face-to-face stacked assemblies, as confirmed by TEM (Figure 2a–f and Figures S2–S5). The injection of cesium oleate triggered immediate nucleation, producing nanosheets that spontaneously organized into well-ordered superlattices within 30–60 s. Aliquots were taken at 10, 20, 30, and 60 s after the injection of cesium oleate into the hot precursor solution to investigate the effect of reaction time on the growth and assembly of CsPbBr_3_ nanosheets (Figure S6). At 10 s, tiny, disordered nanoplates were observed, corresponding to a rapid nucleation burst. After 20 s, partially aligned nanoplates began to appear, marking the onset of self-assembly. After 30 s, larger and more connected ribbon-like domains emerged, indicating enhanced lateral growth and ordering. At 60 s, well-defined, highly ordered superlattice domains were formed, representing the mature stage of assembly. In the main synthesis, the reaction was intentionally stopped after 30 s to allow rapid quenching, enabling the retention of well-defined nanosheet superlattice structures before extensive ripening or aggregation could occur. The ultrathin nanosheets synthesized exhibited bright blue emission under 365 nm UV light (Insert Figure 3f), and their thickness was well below the exciton Bohr diameter of CsPbBr_3_. The exciton Bohr diameter of CsPbBr_3_ was approximately 7 nm. [2,40]. The nanosheets synthesized with thicknesses of 3.35 and 4.05 nm were well below the Bohr diameter and were therefore considered to be in the strong quantum confinement regime, which enhanced their optoelectronic performance. For the thickest nanosheets (~6.5 nm), the effective confinement dimension corresponding to the inorganic core, excluding the surface ligands, indicates that they remain within or near the strong-to-intermediate confinement regime. The surrounding organic ligands (oleic acid and amines) create a low-dielectric environment that enhances Coulomb interactions and effectively increases the confinement strength, further supporting this classification.

**Figure 4:**
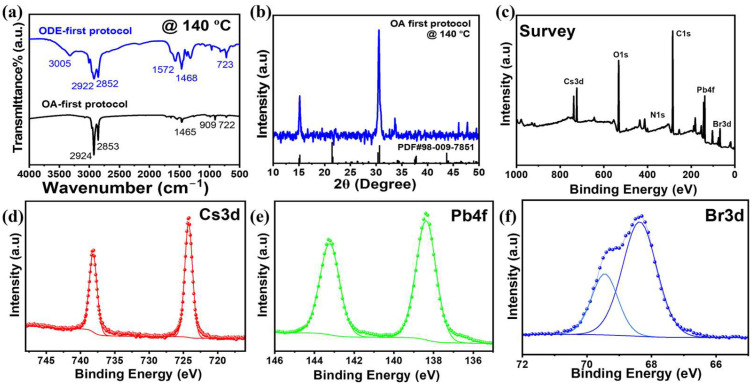
(**a**) FTIR spectra of CsPbBr_3_ nanosheets synthesized using the conventional hot-injection method (ODE-first protocol) and the modified hot-injection method (OA-first protocol). (**b**) XRD pattern of the CsPbBr_3_ nanosheet superlattice synthesized at 140 °C. (**c**–**f**) XPS analysis of the CsPbBr_3_ nanosheet superlattice: (**c**) survey spectrum, (**d**) Cs 3d, (**e**) Pb 4f, and (**f**) Br 3d spectra.

While our approach focuses on controlling the nanosheet size and self-assembly during synthesis, it is instructive to contrast it with other reports on post-synthetic strategies. Previous studies have reduced the size of CsPbBr_3_ nanocrystals by varying the organic acid and amine ligands [10], introducing polar solvents [41], and adding water [42]. In our case, the reaction temperature critically influenced the nucleation and growth dynamics of CsPbBr_3_ nanosheets. At elevated temperatures (150 °C), faster precursor conversion and higher monomer supersaturation drive rapid nucleation, followed by oriented lateral attachment, yielding larger and thicker nanosheets (242.78 ± 75 nm lateral size) (Figure S2e,f and S5). Conversely, at lower temperatures (130 °C, Figure 2b, Figures S2a,b and S3), reduced ion diffusion and slower precursor reactivity limit crystal growth, producing smaller nanosheets with grid-like assemblies of nanosheets with smaller lateral sizes (17.94 ± 0.44 nm) and partially amorphous by-products. These trends reflect a transition from kinetically limited to thermodynamically controlled growth as the temperature increased with increasing time. At 130 °C, the XRD patterns (Figure S8) exhibited broad, featureless backgrounds, indicating the formation of amorphous Pb-Br-rich residues due to incomplete crystallization. When the reaction time was slightly extended at the same temperature, a weak diffraction peak emerged alongside an amorphous background, suggesting the onset of CsPbBr_3_ phase formation. These observations confirm that the low-temperature process yields partially crystallized or disordered products rather than distinct crystalline by-products. Upon increasing the reaction temperature to 140 °C, the amorphous features vanished and sharp diffraction peaks characteristic of orthorhombic CsPbBr_3_ appeared (Figure 4b), demonstrating complete crystallization and the removal of amorphous residues. Strikingly, synthesis at 140 °C provided optimal conditions, yielding nanosheets with ultrathin thickness (4.05 ± 0.09 nm) and face-to-face tiled assemblies with high density. This condition balanced nucleation and growth, allowing for reproducible self-assembly into uniform superlattices. By precisely tuning temperature and limiting the reaction time to 30 s, we demonstrate reliable size-controlled synthesis of CsPbBr_3_ nanosheet superlattices (Figure 2 and Figures S2–S5). 

To further characterize the structural details of these assemblies, high-angle annular dark-field scanning transmission electron microscopy (HAADF-STEM) was employed (Figure 3) on the CsPbBr_3_ nanosheets synthesized at 140 °C. The elemental distributions of Cs, Pb, and Br were further examined using energy-dispersive X-ray spectroscopy (EDS) (Figure 3b–e). EDS mapping revealed that Cs (yellow), Pb (green), and Br (red) were uniformly distributed across the nanosheets, despite minor challenges in focusing on individual nanosheets. These results provide direct evidence for the successful formation of CsPbBr_3_ superlattice nanosheets and confirm that their synthesis can be reliably reproduced at 140 °C. The inset in Figure 3f shows the synthesized solution under 365 nm UV illumination and ambient light.

Fourier-transform infrared (FTIR) spectroscopy was employed to compare the ligand environments of CsPbBr_3_ nanosheets prepared using the conventional ODE-first method and our ligand-mediated protocol (Figure 4a). In the ODE-first synthesis, the weak features at 3005 cm^−1^ and 2922/2852 cm^−1^ correspond to the C–H stretching vibrations of long alkyl chains, while bands at 1572 and 1468 cm^−1^ indicate carboxylate coordination in oleic acid (OA) and other ligands. However, the relatively weak and broadened signals suggest incomplete precursor–ligand binding, which is consistent with the poor regulation of nanosheet growth. In contrast, the ligand-mediated protocol exhibited sharper and more intense vibrational bands, confirming the strong precursor–OA interactions established during the pre-mixing step. The robust C–H stretches and distinct COO^−^/C=O features highlight that OA remains firmly bound, both stabilizing nucleation and supporting self-assembly even after purification, where most ligands have been removed. These results demonstrate that the pre-coordination of ligands enhances reproducibility, yielding uniform nanosheet superlattices with controlled dimensions, in contrast to the irregular assemblies obtained using the ODE-first approach (Figure S1).

X-ray diffraction (XRD) analysis was performed to evaluate the crystallographic structures of the nanosheets synthesized via the ligand-mediated protocol. As shown in Figure 4b, the diffraction pattern of the sample prepared at 140 °C exhibits sharp and well-defined peaks, confirming the high crystallinity of the product prepared at this temperature. All reflections can be indexed to orthorhombic CsPbBr_3_, in agreement with the standard reference pattern (PDF#98-009-7851), verifying the phase purity of the nanosheets. Prominent peaks at 2θ ≈ 15°, 21°, 30°, and 34° correspond to the (100), (110), (200), and (210) planes, respectively, which is consistent with previously reported CsPbBr_3_ perovskite structures. The absence of secondary phases further demonstrates the reliability of ligand-mediated synthesis. The relative intensity of the low-angle peaks suggests a preferred orientation associated with the two-dimensional nanosheet morphology, supporting the tendency of the nanosheets to assemble into ordered layered superlattices. These findings confirm that the modified protocol produces phase-pure, highly crystalline nanosheet superlattices suitable for correlating the structure with optical and electronic performance.

X-ray photoelectron spectroscopy (XPS) was used to analyze the surface chemical states of the CsPbBr_3_ nanosheets (NSs). The survey and high-resolution spectra (Figure 4c–f and Figure S7) confirmed the presence of Cs, Pb, and Br as the primary perovskite constituents. Well-defined doublets were observed for each element, with Cs 3d_5_/_2_ and Cs 3d_3_/_2_ peaks at 724.14 and 738.11 eV, Br 3d_5_/_2_ and Br 3d_3_/_2_ peaks at 68.40 and 69.50 eV, and Pb 4f_7_/_2_ and Pb 4f_5_/_2_ peaks at 138.19 and 143.11 eV, respectively. These values are consistent with Cs^+^, Br^−^, and Pb^2+^ in the CsPbBr_3_ lattice and with those reported in the literature [2,13,43,44]. In addition, the detection of C 1s at 284.80 eV and N 1s at 401.70 and 399.90 eV (Figure S7) reflects the presence of organic ligands, confirming successful surface passivation. Notably, the precise position of the Pb 4f_7_/_2_ peak at 138.19 eV indicates strong Pb–Br interactions, suggesting a bromide-rich surface that passivates halide vacancies and stabilizes the [PbBr_6_]^4−^ octahedral framework. The quantitative surface composition was estimated from the integrated XPS atomic percentages of Cs 3d, Pb 4f, and Br 3d (Table S1). The calculated atomic ratios of Cs:Pb:Br (1.00:0.99:3.39) were close to 1:1:3, consistent with the stoichiometry of CsPbBr_3_, although a slight excess of Br was observed, which may originate from surface bromine enrichment, often reported for halide perovskites. The quantitative results are in good agreement with the elemental composition determined by EDS analysis, confirming the uniform chemical composition of the synthesized CsPbBr_3_ nanocrystals. These results demonstrate that ligand-capped CsPbBr_3_ nanosheets exhibit stable surface chemical states, where the organic ligands enhance the structural integrity, assist in self-assembly, and suppress defect-mediated recombination, thereby improving optoelectronic performance.

**Figure 5:**
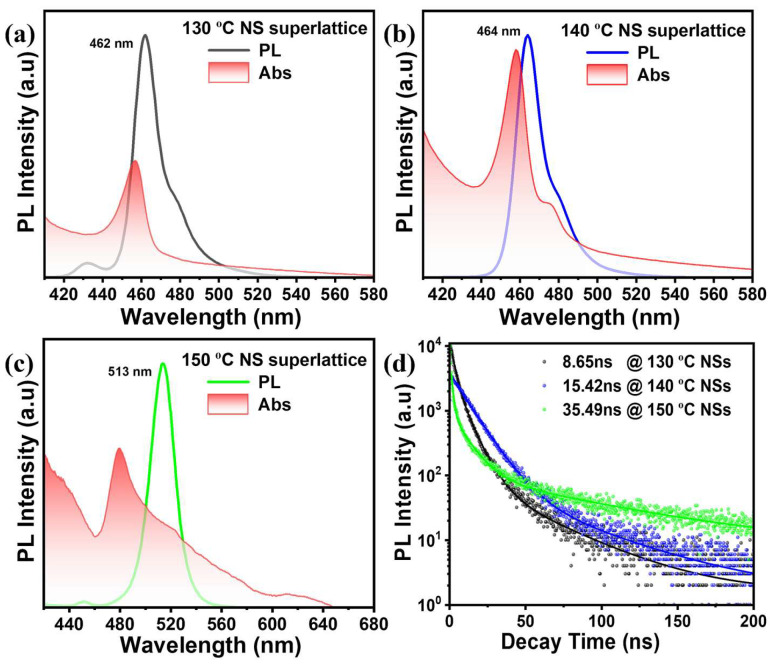
PL and UV-Vis spectra of CsPbBr_3_ NSs superlattice (**a**) 130 °C, (**b**) 140 °C, and (**c**) 150 °C. (**d**) Time-resolved photoluminescence (TRPL) measurements of 2D CsPbBr_3_ NSs superlattice.

Photoluminescence (PL) and UV–Vis absorption spectroscopy were employed to investigate the optical responses of the CsPbBr_3_ nanosheets (NSs) synthesized at 130, 140, and 150 °C (Figure 5 and Table S3). The 130 °C sample, with a lateral size of 17.9 ± 0.44 nm and thickness of 3.35 ± 0.05 nm, exhibited a sharp PL emission at 462 nm, an absorption onset at 457 nm, and a narrow Stokes shift of ~5 nm, which is consistent with strong quantum confinement and efficient radiative recombination. At 140 °C, the nanosheets grew slightly larger (19.02 ± 0.63 nm lateral size, 4.05 ± 0.09 nm thickness) and self-assembled into ordered superlattices, producing a PL peak at 464 nm with absorption at 458 nm and a Stokes shift of ~6 nm. The slight red shift, together with the preserved narrow Stokes shift, reflects the enhanced crystallinity and uniformity achieved during the self-assembly. In contrast, the 150 °C sample displayed a pronounced increase in lateral size (242.78 ± 75 nm) and exhibited a red-shifted PL emission at 513 nm with absorption at 479.16 nm, yielding a significantly larger Stokes shift of ~33.84 nm. This broad shift indicates weakened confinement and stronger exciton–phonon interactions, which is consistent with the reduced overlap between absorption and emission and the partial loss of structural uniformity. These results demonstrate a direct correlation between the synthesis temperature, nanosheet dimensions, and optical response, where controlled growth at 130–140 °C yields confined nanosheets with sharp emission, whereas an elevated temperature of 150 °C induces lateral overgrowth, red-shifted emission, and broadened relaxation pathways, underscoring the importance of temperature regulation for optimizing the performance of nanosheet superlattices in optoelectronic applications.

Time-resolved photoluminescence (TRPL) measurements of 2D CsPbBr_3_ nanosheets synthesized at 130, 140, and 150 °C revealed lifetimes of τ = 8.85, 15.42, and 35.49 ns, respectively, indicating progressively slower carrier recombination with increasing reaction temperatures (Figure 5, Table S2). The nanosheets synthesized at 130 °C (thickness ~3.35 ± 0.05 nm, lateral size 17.94 ± 0.44 nm, PL peak at 462 nm) exhibited a short lifetime, consistent with strong exciton confinement and efficient radiative recombination. At 140 °C, nanosheets with a thickness of 4.05 ± 0.09 nm and lateral size of 19.02 ± 0.63 nm self-assembled into ordered face-to-face superlattices, exhibiting a 464 nm PL peak and an intermediate 15.42 ns lifetime that reflects a balance between confinement and crystal quality. The 150 °C sample, with a larger lateral size and red-shifted PL peak at 513 nm, exhibited the longest lifetime (35.49 ns), consistent with the reduced confinement and slower exciton recombination. The excitonic behavior of the CsPbBr_3_ nanosheet superlattice evolved with temperature, as summarized in Table S3. At 130–140 °C, the emission at 462–464 nm with short lifetimes (8.65–15.42 ns) and small Stokes shifts (5–6 nm) corresponded to free or weakly bound excitons. At 150 °C, the red-shifted emission at 513 nm with an extended lifetime (35.49 ns) and a large Stokes shift (~33.84 nm) indicates the formation of self-trapped excitons (STEs) induced by strong lattice relaxation and exciton localization. The gradual increase in the PL lifetime and Stokes shift with temperature reflects a transition from free to weakly bound and ultimately self-trapped excitons, consistent with earlier reports on lead-halide perovskites and CsPbBr_3_ nanocrystals [45,46]. Although our optical measurements do not directly resolve the charge nature of the excitons, previous studies suggest that in lead-halide systems, both anionic and cationic excitons can coexist via lattice coupling mechanisms. [45,47]. Hence, the observed temperature-dependent transition is attributed primarily to the emergence of self-trapped excitons, rather than a specific charge species. Together, these observations demonstrate how the temperature-dependent dimensional control of CsPbBr_3_ nanosheet superlattices governs exciton dynamics and provides a powerful route for tailoring their performance in optoelectronic applications, such as light-emitting devices and photodetectors.

## 4. Conclusions

This study demonstrates the successful synthesis of 2D CsPbBr_3_ nanosheets (NSs) through a modified ligand-assisted, temperature-controlled hot-injection method (130–150 °C), enabling precise control over their size and optoelectronic properties. Transmission electron microscopy (TEM) revealed uniform 2D NSs, with thicknesses of ~3.35 ± 0.05 nm at 130 °C and 4.05 ± 0.09 nm at the optimal 140 °C and lateral dimensions of 17.94 ± 0.44 nm, 19.02 ± 0.63 nm, and 242.78 ± 75 nm at 130, 140, and 150 °C, respectively. Photoluminescence (PL) spectra showed a clear thickness-dependent emission, shifting from 462 nm at 130 °C to 464 nm at 140 °C and 513 nm at 150 °C. The UV–Vis absorption spectra displayed well-defined onsets with small Stokes shifts for the nanosheet superlattice synthesized at 130 and 140 °C relative to the PL emission, confirming their high crystalline quality and minimal defect states. Notably, the nanosheets self-assembled into ordered superlattices while preserving their intrinsic optical features, underscoring their structural integrity and stability.

These findings highlight temperature and ligand coordination as key parameters for tailoring nanosheet morphology and optical performance. The established synthesis route provides a robust platform for integrating high-quality 2D CsPbBr_3_ superlattices into optoelectronic devices, such as light-emitting diodes and optical sensors, where their size-tunable quantum confinement effects can be harnessed for advanced applications.

## Data Availability

The original contributions presented in this study are included in the article/Supplementary Materials. Further inquiries can be directed to the corresponding author.

## Supplementary Materials

The following supporting information can be downloaded at https://www.mdpi.com/article/10.3390/ma18214885/s1. Figure S1: (a) Schematic illustration of ligand–precursor interactions in the modified protocol, where oleic acid (OA) is introduced and mixed with the precursor prior to the addition of the solvent 1-octadecene (ODE). In this approach, OA ligands bind effectively to the precursors, promoting controlled crystal growth and facilitating the self-assembly of the nanosheets. Representative TEM images of nanosheets synthesized at 140 °C and 150 °C using this method are shown on the right. (b) Schematic diagram of the conventional hot-injection synthesis, in which ODE is added first, followed by the precursors and ligands. In this case, OA interacts weakly with the precursors during the initial reaction stage, resulting in less regulated crystal growth and partial ligand loss during purification. The corresponding TEM images (right) reveal poor growth control and reduced nanosheet self-assembly into superlattices. Figure S2: TEM images (top) and corresponding lateral size distributions (bottom) of 2D CsPbBr_3_ nanosheet superlattices synthesized at different temperatures: (a,b) 130 °C, (c,d) 140 °C, and (e,f) 150 °C. Figure S3: (a–c) TEM image of 2D CsPbBr3 NSs superlattice at to 130 °C. Figure S4: (a–c) TEM image of 2D CsPbBr3 NSs superlattice at to 140 °C. Figure S5: (a–c) TEM image of 2D CsPbBr3 NSs superlattice at to 150 °C. Figure S6: TEM image of 2D CsPbBr_3_ nanosheet superlattices showing the morphology and self-assembly of nanosheets at different reaction times, highlighting the size evolution and formation of ordered superstructures. Figure S7: (a–f) XPS profile of CsPbBr3 NSs superlattice (a) Cs3d, (b) Pb4f, (c) Br3d, (d) C1s, (e) N1s, (f) Survey, elements for CsPbBr3 nanosheets superlattice. Figure S8: XRD patterns of CsPbBr3 nanosheets synthesized at 130 °C with reaction times of 30 s and 120 s. The 30 s sample exhibits a broad, featureless background indicative of amorphous Pb-Br-rich residues, while the 120 s sample shows a weak emerging diffraction peak alongside the amorphous halo, suggesting the onset of CsPbBr3 crystallization under prolonged reaction time. Table S1: Atomic composition of CsPbBr3 determined by XPS. Table S2: Detailed recombination lifetimes determined using an exponential fitting approach. Table S3: Detailed synthesis temperature, Lateral size, Absorption, Photoluminescence and Stoke Shift of nanosheets superlattice.
