# Supplementary material for: Ligand-Mediated, Temperature-Tuned Synthesis of CsPbBr3 Nanosheets for Ordered Superlattice Assembly
Source: Materials (Basel). 2025 Oct 24;18(21):4885. doi: 10.3390/ma18214885 (PMC12608936; doi:10.3390/ma18214885)
Supplement: Supplementary file 1 [file materials-18-04885-s001.zip › materials-3923128-supplementary.pdf]

Supporting Information

Ligand-Mediated, Temperature-Tuned Synthesis of $CsPbBr_3$ Nanosheets for Ordered Superlattice Assembly

Zahir Abdalla, Chengqi Liu, Shefiu Kareem, Xiaoqian Wang, Zisheng Tang, Yong Liu*

[1] State Key Laboratory of Advanced Technology for Materials Synthesis and Processing, Wuhan University of Technology, Wuhan 430070, China; zahiralbashir3@gmail.com (Z.A.); liuchengqi42@163.com (C.L.); shefiu548@hotmail.com (S.K.); 303568@whut.edu.cn (X.W.); tangzs3076@163.com (Z.S).)
* Correspondence: liuyong3873@whut.edu.cn (Y.L.)

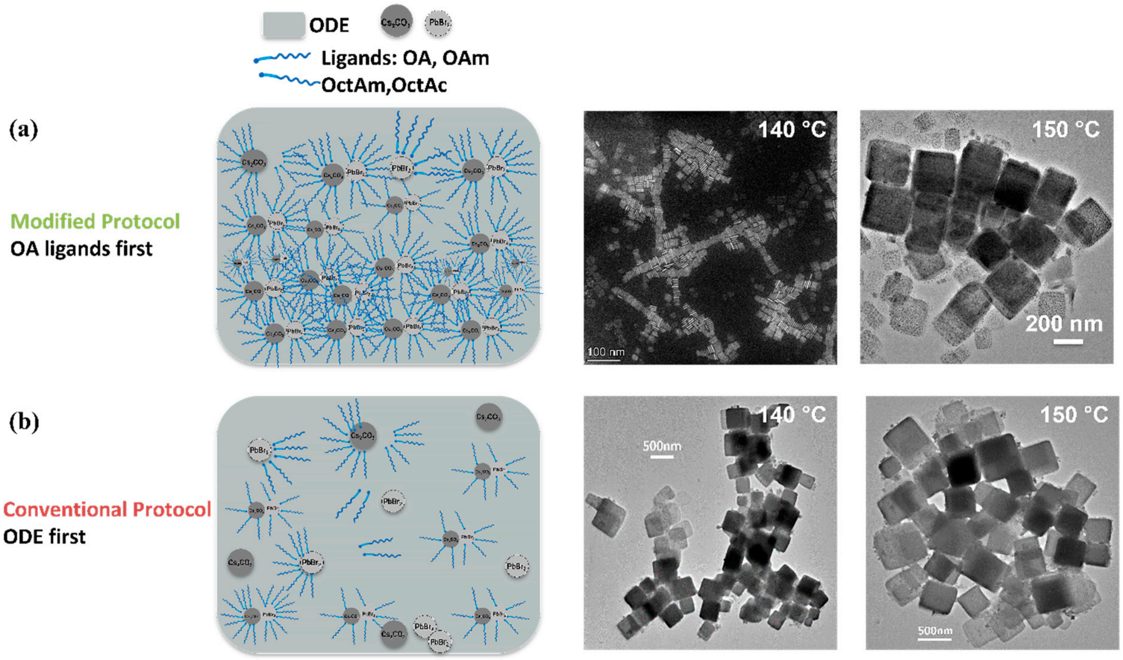

Figure S1. (a) Schematic illustration of ligand–precursor interactions in the modified protocol, where oleic acid (OA) is introduced and mixed with the precursor prior to the addition of the solvent 1-octadecene (ODE). In this approach, OA ligands bind effectively to the precursors, promoting controlled crystal growth and facilitating the self-assembly of the nanosheets. Representative TEM images of nanosheets synthesized at 140 °C and 150 °C using this method are shown on the right. (b) Schematic diagram of the conventional hot-injection synthesis, in which ODE is added first, followed by the precursors and ligands. In this case,

OA interacts weakly with the precursors during the initial reaction stage, resulting in less regulated crystal growth and partial ligand loss during purification. The corresponding TEM images (right) reveal poor growth control and reduced nanosheet self-assembly into superlattices

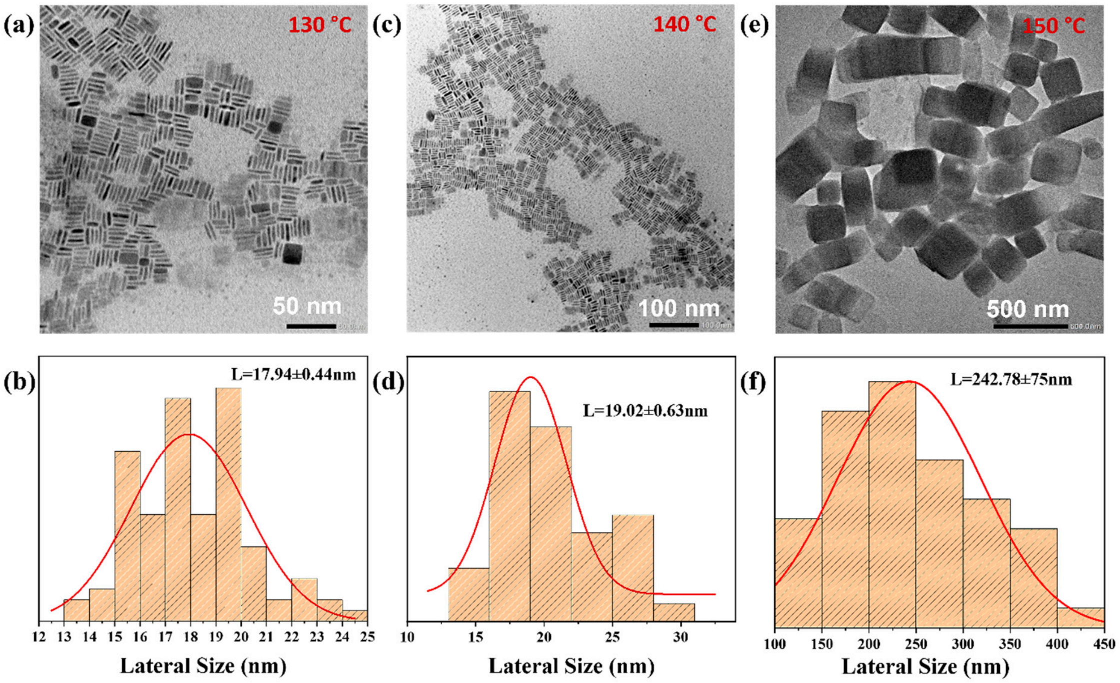

Figure S2. TEM images (top) and corresponding lateral size distributions (bottom) of 2D $CsPbBr_3$ nanosheet superlattices synthesized at different temperatures: (a,b) 130 °C, (c,d) 140 °C, and (e,f) 150 °C

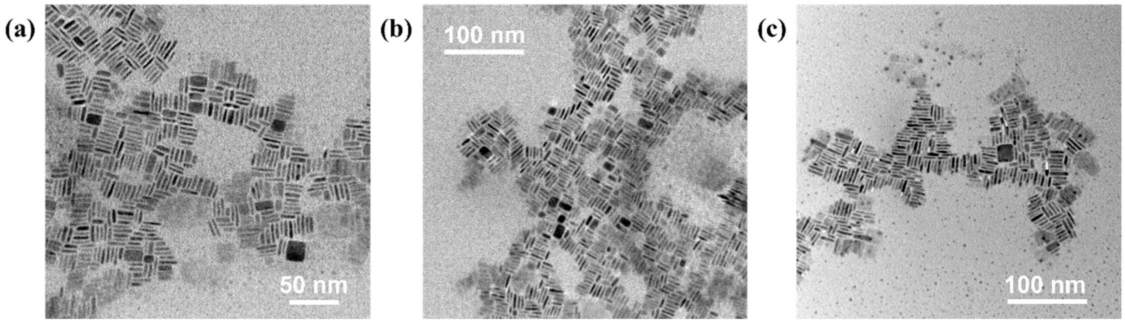

Figure S3. (a-c) TEM image of 2D $CsPbBr_3$ NSs superlattice at to 130 °C

.

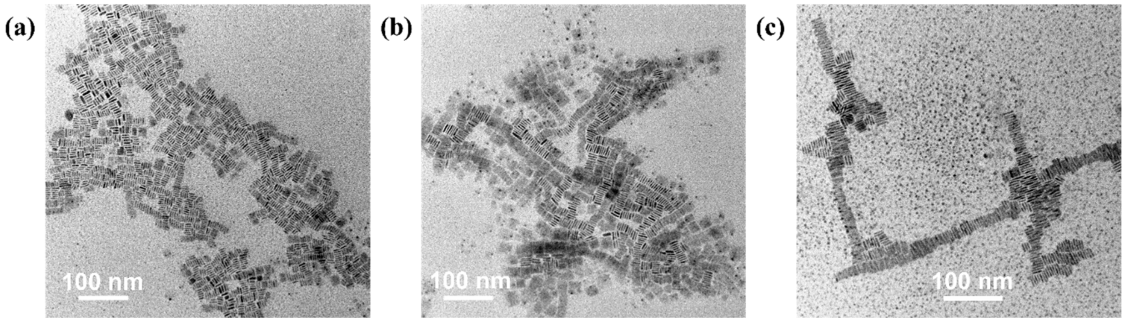

Figure S4. (a-c) TEM image of 2D $CsPbBr_3$ NSs superlattice at to 140 °C

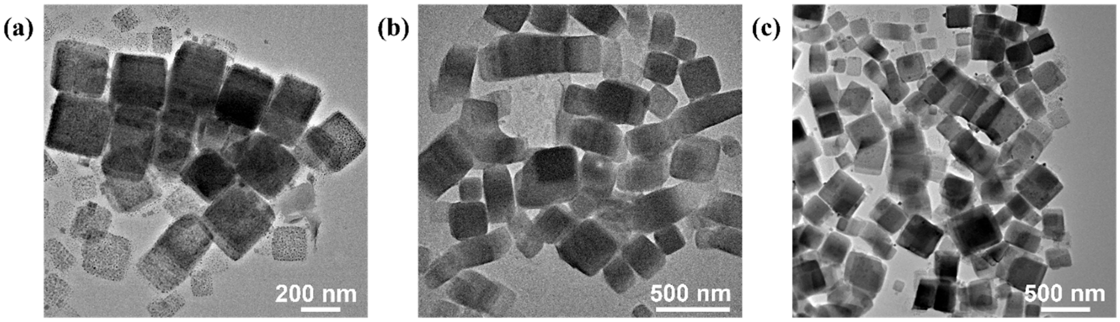

Figure S5. (a-c) TEM image of 2D $CsPbBr_3$ NSs superlattice at to 150 °C

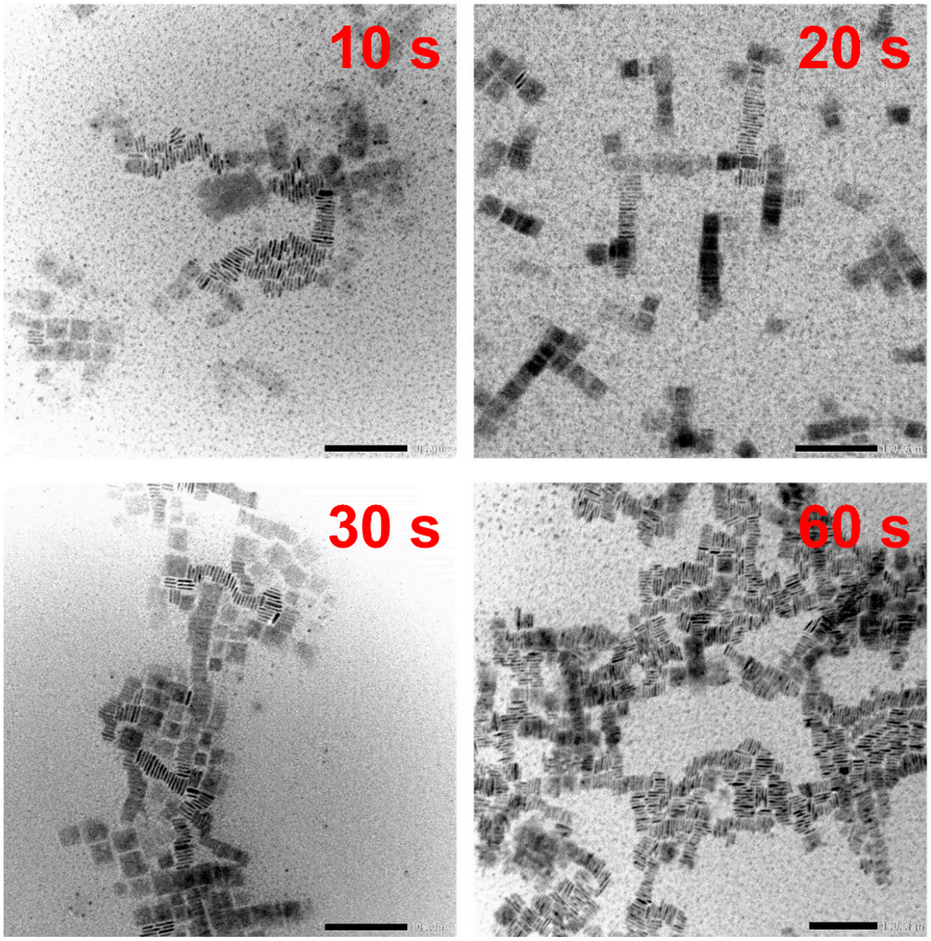

Figure S6. TEM image of 2D $CsPbBr_3$ nanosheet superlattices showing the morphology and self-assembly of nanosheets at different reaction times, highlighting the size evolution and formation of ordered superstructures

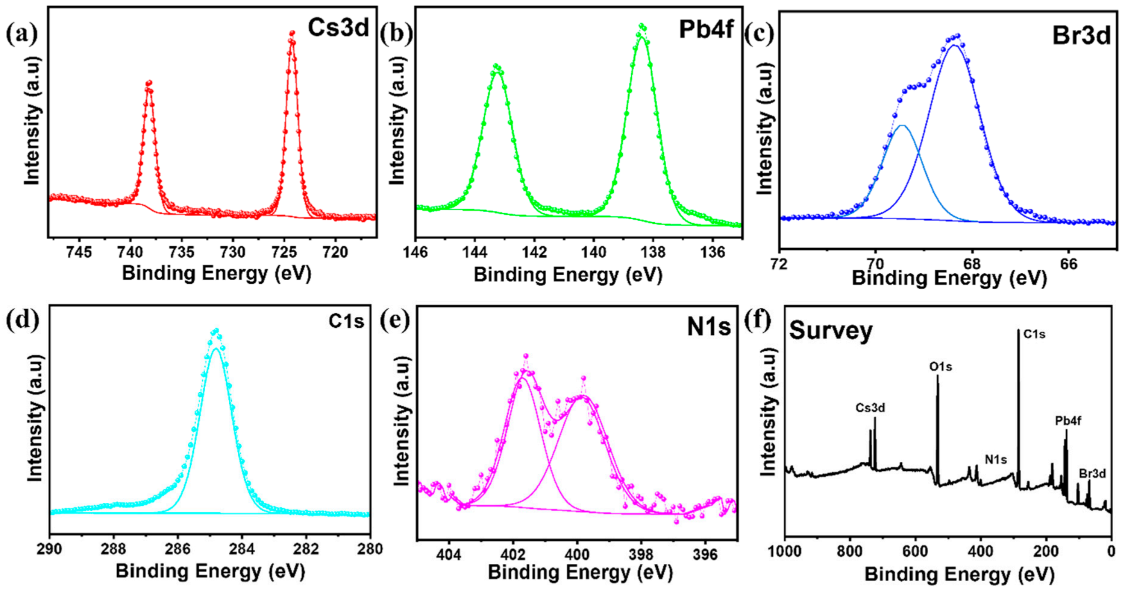

Figure S7. (a-f) XPS profile of $CsPbBr_3$ NSs superlattice **(a)** Cs3d, **(b)** Pb4f, **(c)** Br3d, **(d)** C1s, **(e)** N1s, **(f)** Survey, elements for $CsPbBr_3$ nanosheets superlattice.

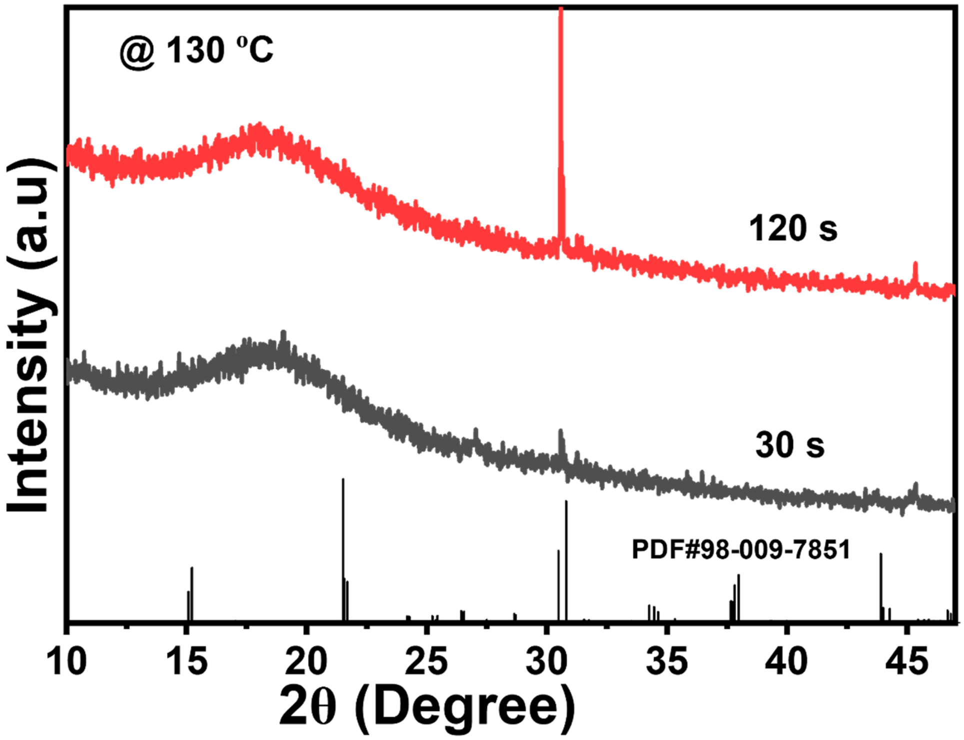

Figure S8. XRD patterns of $CsPbBr_3$ nanosheets synthesized at 130 °C with reaction times of 30 s and 120 s. The 30 s sample exhibits a broad, featureless background indicative of amorphous Pb-Br-rich residues, while the 120 s sample shows a weak emerging diffraction peak alongside the amorphous halo, suggesting the onset of $CsPbBr_3$ crystallization under prolonged reaction time.

Table S1. Atomic composition of $CsPbBr_3$ determined by XPS.

Elements	Atomic % (XPS)	Atomic Ratio
**Cs3d**	20.18	1.00
**Pb4f**	19.92	0.99
**Br3d**	68.45	3.39
**C1s**	2.07	-
**N1s**	1.38	-

Table S2. Detailed recombination lifetimes determined using an exponential fitting approach.

Sample	Γ_1 (ns)	A_1(%)	Γ_2 (ns)	A_2(%)	Γ_3 (ns)	A_3(%)	Γ_{ave} (ns)
**$CsPbBr_3$ @130**	2.75	37.91	8.01	53.26	37.90	8.82	8.65
**$CsPbBr_3$ @140**	11.47	89.29	48.38	10.71	-	-	15.42
**$CsPbBr_3$ @150**	1.24	20.28	9.95	40.32	79.25	39.40	35.49

The time-resolved PL decay curves were fitted using a multi-exponential function:

$$Y = A_1 exp\left({}^{-t}/_{\Gamma_1}\right) + A_2 exp\left({}^{-t}/_{\Gamma_2}\right) + A_3 exp\left({}^{-t}/_{\Gamma_3}\right)$$

The average lifetime Γave was calculated according to:

$$\Gamma_{ave} = \sum A_i \Gamma_i^2 / \sum A_i \Gamma_i$$

Where, Γ1 represents the short-lived component, typically attributed to radiative recombination, while the longer-lived terms (Γ2 for 140 °C and Γ3 for 130 °C and 150 °C) are mainly associated with non-radiative processes. The coefficients A1, A2 and A3 denote the relative weighting of each recombination pathway in the overall decay profile.

Table S3. Detailed synthesis temperature, Lateral size, Absorption, Photoluminescence and Stoke Shift of nanosheets superlattice.

Sample	Lateral size (nm)	Absorption (Abs, nm)	Photoluminescence (PL, nm)	Stoke Shift (nm)	Exciton/Trap State
**$CsPbBr_3$ @130**	17.9 ± 0.44	457	462	5	Free or weakly bound exciton
**$CsPbBr_3$ @140**	19.02 ± 0.63	458	464	6	Weakly/ Intermediate trapping
**$CsPbBr_3$ @150**	242.78 ± 75	479.16	513	33.84	Self-trapped exciton
